# A reaction–diffusion mechanism influences cell lineage progression as a basis for formation, regeneration, and stability of intestinal crypts

**DOI:** 10.1186/1752-0509-6-93

**Published:** 2012-07-31

**Authors:** Lei Zhang, Arthur D Lander, Qing Nie

**Affiliations:** 1 Department of Mathematics, University of California, Irvine, CA, 92697, USA; 2 Department of Development and Cell Biology, University of California, Irvine, CA, 92697, USA; 3 Center for Complex Biological Systems, University of California, Irvine, CA, 92697, USA; 4 Center for Mathematical and Computational Biology, University of California, Irvine, CA, 92697, USA

## Abstract

**Background:**

Colon crypts, a single sheet of epithelia cells, consist of a periodic pattern of stem cells, transit-amplifying cells, and terminally differentiated cells that constantly renew and turnover. Experimental evidence suggests that Wnt signaling promotes and regulates stem cell division, differentiation, and possible cell migrations while intestinal BMP signaling inhibits stem cell self-renewal and repression in crypt formation. As more molecular details on Wnt and BMP in crypts are being discovered, little is still known about how complex interactions among Wnt, BMP, and different types of cells, and surrounding environments may lead to de novo formation of multiple crypts or how such interactions affect regeneration and stability of crypts.

**Results:**

We present a mathematical model that contains Wnt and BMP, a cell lineage, and their feedback regulations to study formation, regeneration, and stability of multiple crypts. The computational explorations and linear stability analysis of the model suggest a reaction–diffusion mechanism, which exhibits a short-range activation of Wnt plus a long-range inhibition with modulation of BMP signals in a growing tissue of cell lineage, can account for spontaneous formation of multiple crypts with the spatial and temporal pattern observed in experiments. Through this mechanism, the model can recapitulate some distinctive and important experimental findings such as crypt regeneration and crypt multiplication. BMP is important in maintaining stability of crypts and loss of BMP usually leads to crypt multiplication with a fingering pattern.

**Conclusions:**

The study provides a mechanism for de novo formation of multiple intestinal crypts and demonstrates a synergetic role of Wnt and BMP in regeneration and stability of intestinal crypts. The proposed model presents a robust framework for studying spatial and temporal dynamics of cell lineages in growing tissues driven by multiple signaling molecules.

## Background

The colonic crypt, a basic functional unit of the intestine, is made up of a single sheet of columnar epithelial cells, which form finger-like invaginations into the underlying connective tissue of lamina propria [1]. A human colon that consists of millions of crypts undergoes continual self-renewal and the intestinal epithelium is completely renewed within 3–5 days in humans [2]. Evidence has pointed to the location of the stem-cell population at the base of the crypt, within the stem-cell niche, formed by the stem cells themselves and mesenchymal cells that surround the crypt base [3]. Stem cells are thought to feed a spatial compartment above the crypt base where most cell proliferation occurs. This part of crypt is thought to house the transit-amplifying (TA) cells that may be committed to one or more cell lineages. The TA cells migrate upwards along the crypt wall toward the luminal surface to give rise to terminally-differentiated (TD) cells that either undergo apoptosis and/or are shed into the lumen and transported away [4].

Wnt signaling controls stem behaviors, maintains stem cell habitus, and regulates cell migration and differentiation [5-7]. Evidence shows that Wnt signaling, through the transcription factor *β*-catenin, is spatially graded along the crypt axis and this spatially-dependent signal provides a possible position-dependent regulation and control of cell proliferation, differentiation and death [8].

Another important player in regulating stem cells and crypt dynamics is bone morphogenetic proteins (BMPs), which are part of the transforming growth factor *β* (TGF-*ß*) superfamily of morphogenetic proteins. Several experiments suggest that BMP signaling is essential for full maturation of the intestinal secretory cell lineage *in vivo*[9], regulates apoptosis of mature colonic epithelial cells [10], and inhibits intestinal stem cell self-renewal through suppression of Wnt-*ß*-catenin signaling [11].

Mathematical models for populations of different cell types and their interactions that do not consider spatial effects have been used to explain premalignant growths and the long lag phases in tumor growth [12,13]. In a spatial model, the crypt has been described as a cylindrical tube in which individual cells of different types were connected using linear spring forces to determine cell migration and organization [14]. This model has been extended to include spatial variation in cell proliferation and adhesion capability that might be caused by graded spatial Wnt signaling along the crypt axis [15], leading to clonal expansion and niche succession to occur. Recently, a continuum model that accounts for cell motion and cell proliferation has been developed for mutations in the Wnt pathway and mutant cell invasion in the crypt [16]; and simulations of an agent-based dynamic model has suggested that reversibility and flexibility of cellular decisions are key elements of robust tissue organization of the intestine [17].

In the existing models, crypts are usually regarded as prescribed and fixed units, and focuses of study have mainly been on dynamics of different types of cells and their mutants within single crypt. Interesting questions at hand include what minimal regulatory interactions and components may give rise to spontaneous formation of multiple crypts with a periodic pattern of stem cells, TA cells, and TD cells along the crypt direction as observed in experiments? What emerging crypt dynamics may arise due to Wnt signaling with regulation from BMP? Because emanation of multiple crypts is directly related to crypt homeostasis [18] and multiplication [9], a model that intimately integrates stem cells and other types of cells with Wnt signaling and BMP signaling for the formation of multiple crypts may be employed for the colonic crypt organization, patterns of stem cell divisions, niche succession, and clonal conversion as evident in the experimental data.

Here, we present a mathematical model that couples a two-stage cell lineage of progenitor cells, which are considered as a sum of stem cells and various types of TA cells, and TD cells with Wnt and BMP signals to study spontaneous formation of multiple crypts. We investigate pattern formation of Wnt signaling arising through interactions among Wnt and a possible Wnt inhibitor (for example, Dickkopf proteins [18]), and their effects on cell proliferation [19-21]. Unlike a classical Turing pattern arising in a fixed or a growing domain [21], Wnt, Wnt inhibitor, and BMP molecules are produced at each cell of a growing tissue and the cell replication probability is controlled by Wnt and BMP signaling through complex feedback regulations and spatial interactions.

Our computational exploration of the model suggests that Wnt patterning driven by the mechanism of short-range activation and long-range inhibition from the Wnt inhibitor with additional modulation from BMP can spontaneously result in formation of multiple crypts. Once a stable multiple-crypt system is established, the loss of crypt or progenitor cells can be regenerated through the regulations and components presented in the model without invoking additional mechanisms. Our computation and analysis also indicate that a loss of BMP may result in formation of more crypts (i.e. crypt multiplication) with a stable configuration; however, stronger Wnt signaling for a system without BMP may weaken such stability, leading to unbounded growth of multiple crypts as seen in experiments [22]. The overall model that couples cell lineages and spatial dynamics of Wnt and BMP along with their regulations on cell proliferation and differentiation shows complex roles of Wnt and BMP in crypt generation, stability and maintenance.

## Results and discussion

### A multiple-crypt model that couples a cell lineage with Wnt and BMP activities

**Figure 1:**
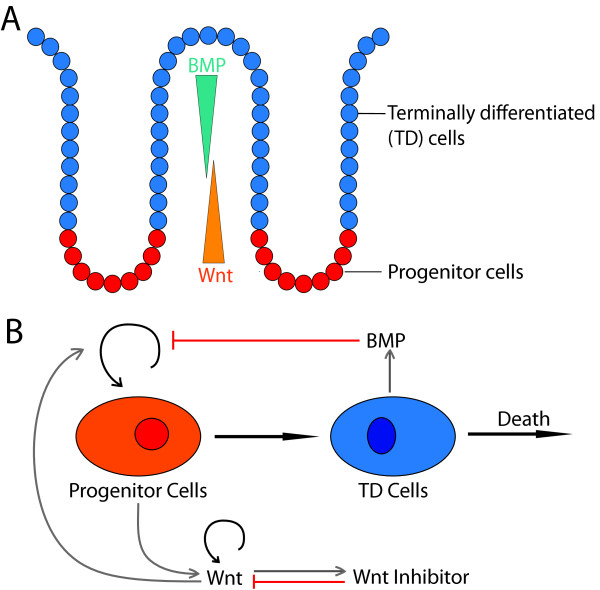
**A schematic diagram of a two-stage cell lineage model. (A)** A cartoon of one colonic crypt and the relative location of cells at different lineage stage adapted from [7,24]. **(B)** A diagram of an un-branched cell lineage that starts with progenitor cells, regarded as a combination of stem cells and transient amplifying cells, leading to terminally differentiated (TD) cells that may undergo death. Replication of progenitor cells may be enhanced by Wnt signaling and be inhibited by BMP signaling. Wnt and BMP are produced by progenitor cells and TD cells, respectively. Wnt has an autoregulation feedback and produces Wnt inhibitor which in turn represses Wnt.

The epithelial cells lining the crypts include stem cells, TA cells and TD cells. Take C_0_ (*s,t*) as the cell density of progenitor cells, i.e. a combination of stem and TA cells, denoted as progenitor cells, along the crypt direction, *s*, and C_1_ (*s,t*) as the density of TD cells (Figure 1A). Similar to a previous one-dimensional model for tissue stratification [23], *C*_0_ and *C*_1_ are governed by

(1) $$\left\{ \begin{array}{l} \frac{\partial C_{0}}{\partial t}+\frac{\partial (VC_{0})}{\partial s}=v_{0}(2p_{0}-1)C_{0},\\ \frac{\partial C_{1}}{\partial t}+\frac{\partial (VC_{1})}{\partial s}=v_{0}(2(1-p_{0})C_{0})-d_{1}C_{1},\\ \frac{\partial V}{\partial s}=v_{0}C_{0}-d_{1}C_{1}\text{.} \end{array}\right.$$

Here, *p*_0_ represents the replication probability of progenitor cells, and parameter *v*_0_ is the reciprocal of the cell cycle length scaled by ln 2; *d*_1_ is the removal rate for TD cells and it may be spatially regulated (see Section I in Additional file 1 for formulation); *V*(*s*, *t*) is the velocity of cells at crypt location, *s*, enabling a constant density of total cells at any spatial location, i.e. *C*_0_ + *C*_1_ = 1, up to a normalization [23].

In colonic crypts, epithelial cells at different stages of the lineage may be exposed to different levels of signaling. The spatial heterogeneity of cell dynamics arises from the fact that the replication probability of progenitor cells is spatially and temporally regulated by secreted molecules such as Wnt and BMP, which may provide robust controls for homeostasis and the spatial arrangement of cells. Experimental evidence suggests that the proliferation rate of progenitor cells is enhanced by Wnt activity [5,6] and inhibited by BMP signaling [11,25]. This regulatory effect from the secreted molecules produced by progenitor and TD cells may be modeled through a Hill equation on the replication probability

(2) $$p_{0}={\bar{p}}_{0}\frac{{\left(\gamma_{W}[Wnt]\right)}^{n}}{\left(1+{\left(\gamma_{W}[Wnt]\right)}^{n}+{\left(\gamma_{B}[BMP]\right)}^{m}\right)}\text{.}$$

Here, [Wnt] and [BMP] represent the concentrations of molecules Wnt and BMP, respectively; ${\bar{p}}_{0}$ is the maximal replication probability; *γ*_*w*_ and *γ*_*B*_ are reciprocals of the corresponding half maximal effective concentrations (EC50), and *m* and *n* are corresponding Hill exponents.

Because the spatial distribution of progenitor and TD cells from the basal to the apical surface may intimately depend on the spatial distribution of the diffusive molecules Wnt and/or BMP, we consider spatial and temporal dynamics of Wnt and BMP as well as a possible Wnt inhibitor [26]. Consistent with experimental observations [5,6,11,25], Wnt is assumed to be produced by progenitor cells and BMP is produced by TD cells; Wnt has an auto-regulation feedback [7]; and Wnt inhibitor is assumed to be positively regulated by Wnt, as suggested by the Wnt system in hair follicle [20]. The overall dynamics of Wnt, Wnt inhibitor, and BMP (Figure 1B) can be described by a system of convection-reaction–diffusion equations

(3) $$\left\{ \begin{array}{l} \frac{\partial [Wnt]}{\partial t}+\frac{\partial (V[Wnt])}{\partial s}=D_{W}\frac{\partial^{2}[Wnt]}{\partial s^{2}}+\mu_{0}C_{0}+\\ \frac{\alpha_{W}}{\left(1+{\left(\beta_{W}[Wnt]\right)}^{-n_{W}})(1+{\left(\beta_{I}[In]\right)}^{m_{W}}\right)}-d_{W}[Wnt],\\ \frac{\partial [In]}{\partial t}+\frac{\partial (V[In])}{\partial s}=D_{I}\frac{\partial^{2}[In]}{\partial s^{2}}+\frac{{\bar{\alpha }}_{W}}{1+{\left({\bar{\beta }}_{W}[Wnt]\right)}^{-n_{I}}}-d_{I}[In],\\ \frac{\partial [BMP]}{\partial t}+\frac{\partial (V[BMP])}{\partial s}=D_{B}\frac{\partial^{2}[BMP]}{\partial s^{2}}+\mu_{1}C_{1}-d_{B}[BMP]\text{.} \end{array}\right.$$

Here, each type of molecule assumes individual effective diffusion rates *D*_*W*_, *D*_*I*_, *D*_*B*_. The removal of molecules due to degradation or binding with other molecules is assumed to be linearly proportional to the concentration of each molecule, with a rate constant *d*_*W*_, *d*_*I*_, *d*_*B*_. The synthesis of Wnt and BMP is assumed to be proportional to the density of the cell types that produce Wnt and BMP with rates *μ*_0_, *μ*_1_. The coefficient *α*_*W*_ is used to describe the rate of self-enhanced Wnt activity and _*W*_ is the synthesis rate of Wnt inhibitor. *β*_*W*_*β*_*I*_, and _*W*_ are reciprocals of the EC50 which reflect the strength of feedbacks of Wnt and Wnt inhibitor. *n*_*W*_, *m*_*W*_, and *n*_*I*_ are Hill coefficients. The choice of the biochemical parameters, such as diffusion coefficients, is drawn from previous cell lineage models of similar molecules and from experimental approximations (for instance, [16,23,27]).

Typically, the time scale of the cell cycle length is days, whereas that of the molecule interactions is hours [28]. Thus, the dynamics of molecules may quickly reach a quasi-steady state within a cell cycle. In the numerical simulations, we compute the time evolution of the cells in Eq (1) using the longer time scale based on the cell cycle length, and at each time step we solve the quasi-steady states of the molecules in Eq. (3) for computational efficiency [23].

Because the connected multiple crypts in the intestine typically exhibit periodic behavior if the interests of study is within a domain containing a small number of crypts [29], we assume all quantities have periodic boundary conditions within the one-dimensional direction,*s*. For a natural representation of crypts of curving shape, we employ a gradient flow of energy functional [30] to model the spatial and temporal evolution of crypts in a two-dimensional space:

(4) $$\frac{d(x(s,t),y(s,t))}{dt}=-\nabla_{\left(x,y\right)}(E(x,y,C_{0}(s,t),C_{1}(s,t))\text{.}$$

Here (*x*(*s*, *t*), *y*(*s*, *t*)) is a description of crypts in the two-dimensional space as a function of the one-dimensional variable *s*and time*t*. The energy functional *E* is a functional of cell densities, and it can be regarded as a phenomenological and simple description of overall mechanical and other effects exerting on the crypt from its surrounding environment as cells proliferate and differentiate along the crypt direction. The energy functional is made in a form such that locations of progenitor cells correspond to the minimum energy of *E*to enable an overall spatial distribution of different cells in each crypt consistent with experimental observations. One simple form of such an energy functional is

(5) $$E(x,y,C_{0})={\left(y+\max(C_{0})(\tanh(\frac{d(C_{0})-|x-\arg\max_{x}(C_{0}(x))|}{\epsilon_{0}})+1)/2-b\right)}^{2}\text{,}$$

where *d*(*C*_0_) is the diameter of crypt, which is chosen as a scale of integration of the progenitor cells along a crypt; *ε*_0_ represents the sharpness of the crypt shape; and *b*is a constant determining the starting point of the crypt that does not affect length or dynamics of the crypt.

Because the dynamics of crypt morphology changes according to Eq. (4), the total length of the crypt may vary over time, affecting patterns of cells and molecules. To investigate the effect of a growing domain, we include the dynamic length of the domain in the model *L*_max_(*t*) computed by

(6) $$L_{\max}(t)=\int_{a}^{b}\sqrt{x_{s}{}^{2}(s,t)+y_{s}{}^{2}(s,t)}ds\text{,}$$

in which the crypt(*x*(*s*, *t*), *y*(*s*, *t*)) is governed by Eq. (4) and *a*, *b* are the two fixed end points of the domain, taken as (−0.1, 0) and (0.1, 0) in our simulations. Therefore, the model consisting of Eqs. (1–4) is simulated in a growing domain with a dynamic length *L*_max_(*t*) (see Section I of Additional file 1 for the model formulation in a growing domain).

### A Turing pattern of Wnt signaling determines spatial distribution of progenitor and TD cells

We first study how the interaction between the short range of Wnt activation and the long range of Wnt inhibition occurring in a domain of proliferating cells may generate a Turing pattern [31] that in turn affects spatial distribution of progenitor and TD cells.

**Figure 2:**
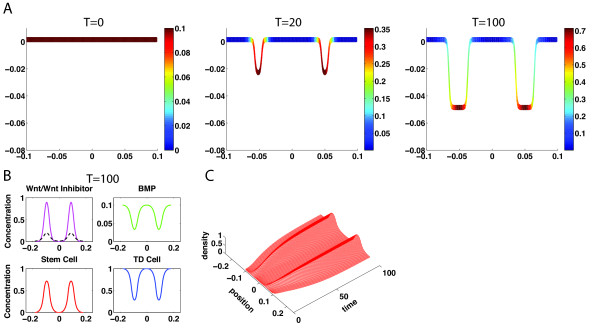
**Spatial distributions of progenitor cells and the crypt growth are driven by a Turing pattern of Wnt signaling. (A)** Dynamics of the crypt growth colored in progenitor cell density at three different times. The solution is close to the steady state at *T* = 100. **(B)** Concentrations of Wnt, Wnt Inhibitor, BMP, progenitor cells, and TD cells along the crypt direction of *s* near the steady state at *T* = 100 (Wnt – magenta solid line, Wnt Inhibitor – black dash line). **(C)** Time evolution of the progenitor cells. Parameters used are listed in Additional file 1: Table S1 and Table S2.

Starting with uniform distributions of progenitor and TD cells along with only low and fluctuated levels of Wnt signals without any initial presence of BMP and Wnt inhibitor, the system can achieve a steady state spatial pattern of cells with progenitor cells localizing in the bottom of crypt and the TD cells residing in the top and majority of the crypt (Figure 2A). From simulations of Eqs. (1–4), Wnt activity quickly adapts a Turing pattern from an initially small random perturbation of Wnt before cells develop into a heterogeneous pattern (Figure 2A at T = 20, 100). Because Wnt signaling positively regulates the replication probability of progenitor cells, progenitor cells then accumulate at locations with high concentration of Wnt, corresponding to two peaks of Wnt pattern (Figure 2B). The spatial distributions of Wnt, Wnt inhibitor, and BMP clearly show a classical Turing patterning in one-dimensional direction along the crypt direction, driving spatial distribution of progenitor cells and TD cells along the crypt direction with a specific crypt shape governed by Eq. (4) (Figure 2A). The temporal dynamics of progenitor cell density shows the expansion of the domain caused by crypt growth (Figure 2C).

Linear stability analysis suggests that Wnt inhibitor must adapt rapidly to dynamics of Wnt in order for the system to form a stable heterogeneous pattern in Wnt and Wnt inhibitor (see Section II in Additional file 1). Specifically, the ratio of the two removal rates, *d*_*I*_ for Wnt inhibitor and *d*_*W*_ for Wnt, i.e.,dIdW, must satisfy a condition for the occurrence of a Turing instability. In addition, the ratio of the two diffusion coefficients between Wnt inhibitor and Wnt and the ratio of the corresponding removal rates are two other important non-dimensional parameters and must satisfy a necessary relationship in order for the system to develop patterns (Additional file 1: Figure S1).

**Figure 3:**
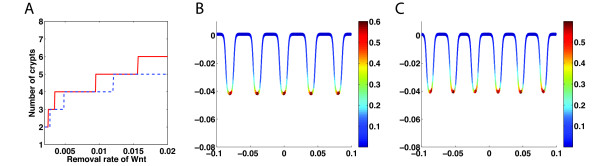
**The number of crypts at the steady state depends on removal rate of Wnt. (A)** The number of crypts at steady state as the removal rate of Wnt is varied for two different initial distributions of Wnt (blue dash line and red solid line). For example, at the same Wnt removal rate of *d*_*W*_ = 0.02*s*^−1^, there are five crypts **(B)** or six crypts **(C)** at steady state starting from two different initial conditions (see Additional file 1: Figure S2 for initial conditions). Other parameters used for the simulation are in Additional file 1: Table S2.

Because Wnt and BMP regulate cell proliferation, the removal rate of the secreted molecules due to degradation or binding with other molecules may significantly affect dynamics of homeostasis and spatial pattern of cells in the intestine. Simulations show that increasing of the equal removal rate of Wnt and Wnt inhibitor usually leads to creation of more crypts (Figure 3A). As generally seen in the formation of Turing patterns, the initial distribution of Wnt plays an important role in creating the steady state patterns due to the existence of multiple steady state solutions of reaction–diffusion equations. The number of crypts can be different at the same high removal rate of Wnt for different initial distributions. For example, a 10-fold increase of removal rate of Wnt results in an increasing number of crypts from two to either five (Figure 3B) or six (Figure 3C) by using two different initial conditions (see Additional file 1: Figure S2). Such Turing patterning is robust to initial fluctuated distributions of progenitor cells.

**Figure 4:**
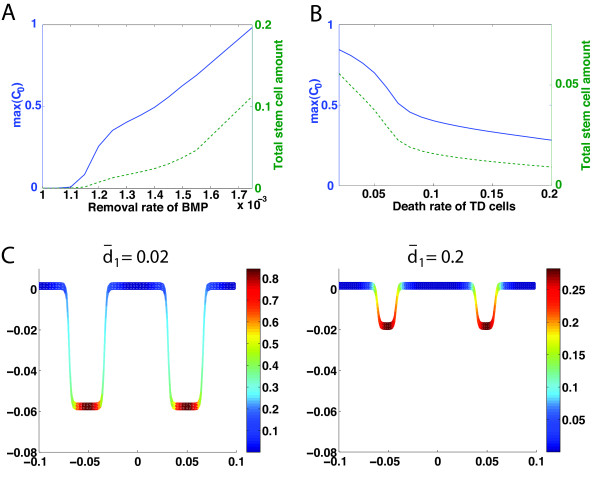
**Maximal density and total amount of progenitor cells on a crypt as functions of removal rate of BMP or maximal death rate of TD cells. (A)** Blue: removal rate of BMP versus maximal density of progenitor cells (max (*C*_0_)); green: removal rate of BMP versus total amount of progenitor cells. **(B)** Blue: maximal death rate of TD cells (${\bar{d}}_{1}$) versus maximal density of progenitor cells (max (*C*_0_)); green: maximal death rate of TD cells versus the total amount of progenitor cells. **(C)** Spatial distribution of progenitor cells and the spatial crypt pattern at ${\bar{d}}_{1}=0.02$ and 0.2. Other parameters used for the simulation are in Additional file 1: Table S2.

The effects of the BMP removal rate are found to be different from those of the Wnt/Wnt inhibitor removal rate. Typically, increasing the BMP removal rate leads to an increase in the maximum density of progenitor cells (denoted by max (*C*_0_)) and the total amount of progenitor cells (Figure 4A); however, unlike the removal rate for Wnt, it does not affect the number of crypts. In particular, a low BMP removal rate can result in crypt extinction (e.g. for *d*_*B*_ < 1.1 × 10^−3^*s*^−1^ in Figure 4A), because BMP inhibits the replication probability of progenitor cells and low BMP removal rate implies stronger BMP signals in the system. By the same token, a high BMP removal rate can lead to the unbounded growth of progenitor cells (e.g. for *d*_*B*_ > 1.75 × 10^−3^*s*^−1^ in Figure 4v).

Since homeostasis of tissue growth requires a balance between cell proliferation and cell death, the death rate of TD cells may also significantly affect dynamics and the spatial distribution of crypts. Specifically, increasing the death rate of TD cells results in a decrease in maximum density of progenitor cells and loss of the total amount of progenitor cells (Figure 4B). A tenfold increase in the maximal death rate of TD cells, ${\bar{d}}_{1}$, results in a 10% decrease of the replication probability of progenitor cells (Additional file 1: Figure S7) and a near threefold decrease in crypt height (Figure 4C). The relationship between the proliferation of cells and crypt height seems to be consistent with the experimental observation showing that the modest changes in cell proliferation rates can account for the large variation of crypt outputs [32].

### Loss of crypts or progenitor cells can be regenerated through Wnt signaling

Experimental evidence has shown Wnt signaling plays a critical role in regulating progenitor cells, suggesting the possibility that the self-renewal property of progenitor cells might be tightly mediated by Wnt signaling [5]. To investigate this observation, we remove a portion of progenitor cells or all progenitor cells in one crypt to examine the role of Wnt signaling in promoting self-renewal of progenitor cells.

**Figure 5:**
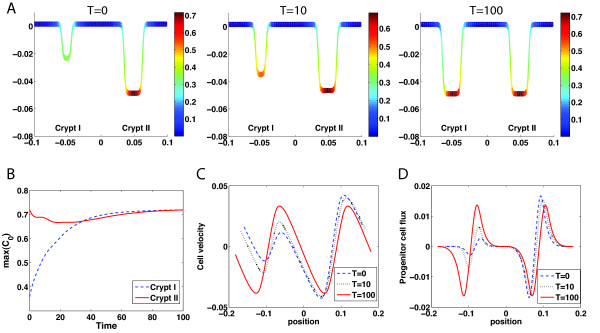
**Loss of progenitor cells or a crypt can be regenerated through Wnt signaling. (A)** At *T* = 0, one half of progenitor cells in Crypt I are removed from the wild-type steady crypt (*T* = 100 in Figure 2A). With the regulation of Wnt signaling, progenitor cells in Crypt I start to repopulate (*T* = 10). Eventually, the original pattern of progenitor cells in Crypt I is regenerated (*T* = 100). **(B)** The maximal density of progenitor cells in Crypt I (blue dash line) and Crypt II (red solid line) as functions of time. Cell velocity, *V*, **(C)** and progenitor cell flux, *VC*_0_, **(D)** are plotted at the time *T* = 0 (blue dash curve), *T* = 10 (black dot curve), and *T* = 100 (red solid curve).

First, we replace half of the progenitor cells in one of the two crypts (denoted as Crypt I) from a two-crypt steady state system (as shown in Figure 2A*T* = 100) by TD cells to enforce the same constant local density of cells along the crypt direction (Figure 5A*T* = 0). The initial concentrations of Wnt, Wnt inhibitor, and BMP are chosen to be the same as the steady state of two-crypt system before removing some of the progenitor cells (corresponding to the density profiles shown in Figure 2C) with all other parameters and conditions in Eqs. (1–4) remaining the same.

Early dynamics show that the density of progenitor cells in Crypt I increases while Crypt I grows in height, because the replication probability of progenitor cells regulated by Wnt and BMP remains high, leading to fast proliferation of progenitor cells. The velocity of cells and the flux of progenitor cells in Crypt I gradually increase (Figure 5C and 5D), as a result, Crypt I becomes larger (Figure 5A*T* = 10). In Crypt II, the dynamics of progenitor cells shows a minor change (Figure 5B). It decreases first due to the movement of cells (Figure 5C), and then increases as the progenitor cells produce more Wnt to enhance their replication probability. Eventually, two crypts are recovered to regain the original two-crypt steady state (Figure 5A*T* = 100).

To further investigate the potential mechanism of the regeneration of progenitor cells, we study the case in which progenitor cells in Crypt I are totally removed and replaced by TD cells from the two-crypt steady state (Additional file 1: Figure S3A *T* = 0) with the rest of conditions and parameters remaining the same as the wild-type ones (as in Figure 2). In this case, although the progenitor cell density at the tip of Crypt II rapidly decreases by ~30% within a short period of time, the progenitor cell density at the tip of Crypt I is at a very low level (100 times lower than the progenitor cell density at the tip of Crypt II (Additional file 1: Figure S3B)), and the flux of progenitor cells is near zero (Additional file 1: Figure S3D). Unlike the case with a partial loss of progenitor cells, the first group of progenitor cells appearing in Crypt I must be physically relocated from Crypt II through cell movement (Additional file 1: Figure S3C) due to the cell proliferation. Only when a small amount of progenitor cells start to accumulate in Crypt I, a Turing pattern of Wnt starts to develop and dominate the dynamics (Additional file 1: Figure S3A *T* = 200). Ultimately, the progenitor cells in Crypt I are regenerated and the two-crypt steady state is recovered (Additional file 1: Figure S3A *T* = 300), similar to the case with the partial loss of progenitor cells. It is important to note that if the migration of a very small number of stem cells (e.g. single stem cell) is responsible for regeneration of the new crypt, a continuum model may not be sufficient for predicting the mechanisms of regeneration, and discrete-cell models that can incorporate stochastic effects are needed [27].

### BMP maintains stability of crypts and loss of BMP may result in crypt multiplication

Experimental data shows that a loss of BMP signaling (through knockout of BMP receptors) in the intestine leads to multiplication and fission of crypt units [9], suggesting that BMP signaling is essential for maintaining the equilibrium and full maturation of the intestinal secretory cell lineage [9]. We hypothesize that BMP is produced by TD cells and then negatively regulates the replication probability of progenitor cells based on previous experimental observations [11,25]. Unlike Wnt, which promotes the generation of progenitor cells, we assume that BMP suppresses the production of progenitor cells by inhibiting the replication probability of the progenitor cells. To investigate the role of BMP, we consider crypt dynamics under different strengths of BMP regulation, including without BMP, in the replication probability.

**Figure 6:**
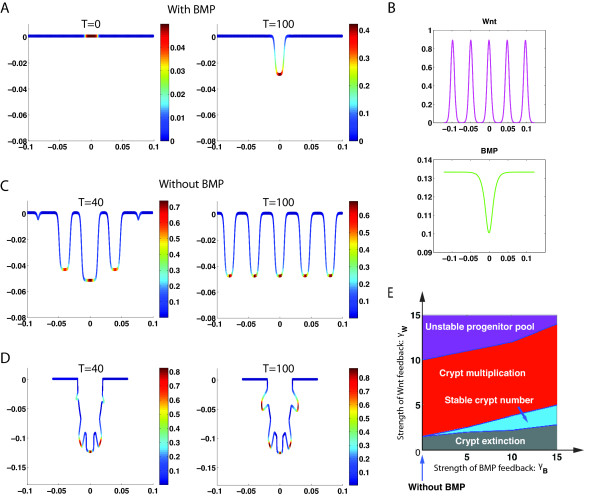
**Dynamics of crypts with or without BMP. (A)** With BMP, starting from a uniform distribution of progenitor cells localized in a small local area of the tissue (*T* = 0), a single crypt is formed near the steady state (*T* = 100) (*μ*_1_ = 2.5 × 10^−4^*s*^−1^*μM*). **(B)** The corresponding patterns of Wnt and BMP near the steady state in the crypt system described in **(A)**. **(C)** If BMP is removed, starting from the single crypt (*T* = 100 in (A)), more small crypts are generated (*T* = 40) and eventually give rise to five identical crypts (*T* = 100). **(D)** Dynamics of crypt multiplication using a different energy function *E* without BMP (see Eq. S1.5 in Additional file 1). **(E)** A phase diagram of crypts in terms of *γ*_*B*_ and *γ*_*W*_ the strengths of feedback for Wnt and BMP, respectively. “Crypt extinction” corresponds to zero density of progenitor cells at the steady state; “Stable crypt number” means the number of crypts at the steady state is equal to the number of the initially localized spots of progenitor cells (e.g. se *T* = 100 in (A)); “Crypt multiplication” stands for the number of steady crypts increases from “stable crypt number” (e.g. *T* = 100 in (C) and (D)); “Unstable progenitor pool” represents the region where the density of progenitor cells in multiple crypts increases without bounds. Other parameters used are listed in Additional file 1: Table S2.

With BMP, starting from a uniform distribution of progenitor cells localized in a small local area of the tissue (Figure 6A*T* = 0), a single crypt is formed at the steady state (Figure 6A*T* = 100). For this case, while Wnt exhibits a pattern of five peaks, BMP exhibits only one peak (Figure 6B) that can dominate the progenitor cell replication probability that Wnt positively regulates and BMP negatively regulates, resulting in single crypt at the steady state.

Starting with this steady configuration of crypts, however, removing BMP from the system as would be the case in a knockout of BMP, more crypts immediately emerge (Figure 6C*T* = 40) and grow, because the replication probability of progenitor cells starts to lose the negative control from BMP and the term is dominated by Wnt signaling. The new system without BMP, now driven by a two-component (i.e. Wnt and Wnt inhibitor) Turing instability, enables crypt multiplication, leading to a new steady pattern of five crypts, with each crypt longer than the original one (Figure 6C*T* = 100). To mimic the branching and fingering morphology observed in the BMP experiments [22], we use a different choice of energy functional *E* (see Eq. (S1.4) in Section I in Additional file 1) to model a potentially different mechanic and biochemical environment surrounding the multiple crypts when BMP is inhibited. As a result, the crypts in the model simulation can branch in a compact form and develop fingers during growth (Figure 6D) as seen experimentally [22].

If initially localized spots of the progenitor cells coincide with the Wnt pattern (Figure 6B), the number of crypts at the steady state for the system with BMP then become the same as the number of initial spots in progenitor cells. The maximal number of crypts is equal to the number of peaks in Wnt pattern, which is five for this case (Figure 6B), despite the fact that more than five spots of progenitor cells may be placed initially (see Additional file 1: Figure S4). An initial uniform distribution of progenitor cells also leads to five crypts at the steady state. Interestingly, starting from the multiple-crypt steady state system with BMP and then removing BMP, the system always evolves to five crypts at a steady state independent of the choice of initial conditions (see Additional file 1: Figure S4).

To further study this behavior, we systematically explore the system by varying *γ*_*w*_ and *γ*_*B*_, which are reciprocal of the EC50s, a measurement of strength of feedback for Wnt and BMP (Figure 6E), using the same localized initial distributions of progenitor cells (Figure 6A*T* = 0). The system exhibits four distinct behaviors based on the strengths of Wnt signaling and BMP signaling (Figure 6E): 1) crypt extinction -- the crypt eventually disappears without any progenitor cells left in the system as long as Wnt regulation is weak; 2) a stable crypt number -- the number of crypts at the steady state equals the number of initially localized spots of progenitor cells if Wnt regulation is moderate with modulation from BMP signaling (e.g. *T* = 100 in Figure 6A); 3) crypt multiplication -- the number of steady crypts increases from the “stable crypt number” when BMP signaling is inhibited/reduced or Wnt signaling is over-expressed (e.g. *T* = 100 in Figure 6C and 6D); and 4) an unstable progenitor pool -- multiple crypts grow with an endlessly increasing amount of progenitor cells if Wnt signaling is strongly expressed even with strong BMP regulation.

In particular, starting from a single steady crypt formed from a system with BMP and then removing BMP (i.e. setting *γ*_*B*_ = 0 in Figure 6E), the system exhibits “crypt multiplication”. Furthermore, starting from multiple crypts that resulted from a system with BMP and then removing BMP, we observe an “unstable progenitor pool” when Wnt regulation is strong enough. In other words, inhibition of BMP signaling results in crypt multiplication with more crypts than the wild-type case; however, only when Wnt signaling becomes sufficiently strong do crypts start to grow in an uncontrollable way, leading to disease, as observed in experiments [22]. Overall, the phase diagram (Figure 6E) shows BMP regulation allows the system to have a larger range of Wnt signals for existence of either a single crypt or multiple crypts at steady state. In a system with BMP, small perturbations of Wnt may not lead to unbounded growth of crypts while the system without BMP can easily have uncontrollable growth of crypts when Wnt is slightly over-expressed, demonstrating the role of BMP in maintaining stability for crypt growth.

### Localized stem cells and exogenous Wnt

**Figure 7:**
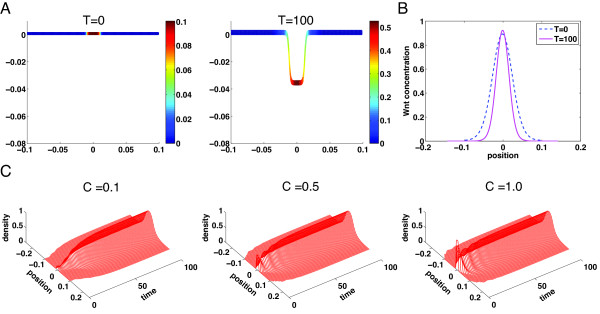
**Spatial distributions of progenitor cells in a growing domain. (A)** Crypt dynamics of progenitor cell density at three different times. A small amount of progenitor cells (C_0_ = 0.1) is initially placed in the center of the domain (1/10 of the length of the entire domain). The solution is close to the steady state at T = 100. **(B)** Comparison of the progenitor cell density along the crypt direction *s* in the fixed domain (blue dash curve) and the growing domain (red solid curve). **(C)** Time evolution of the progenitor cells in single crypt starting with three different initial levels of progenitor cells. The progenitor cells are initially placed at the center of the domain with *C*_0_ = 0.1, 0.5, and 1.0. Parameters in the simulations are listed in Additional file 1: Table S2.

Experiments in culture have suggested that single stem cell can generate crypt-villus structure *in vitro* without a mesenchymal niche [33,34]. In our continuum model, we mimic single stem cell by placing a small amount of progenitor cells only in the small region at the center of the domain initially (Figure 7A at *T* = 0). It is observed this small amount of progenitor cells that release Wnt molecules (Figure 7B), which in turn induce growth, can lead to formation of a full single stable crypt (Figure 7A at *T* = 100). This suggests that Wnt and BMP signals can induce growth and formation of crypts even with a very small number of progenitor cells localized in a small spatial region.

To study how sensitive the final pattern of the crypt depends on the initial density of progenitor cells, we place three different levels of progenitor cells in the same localized spatial region (Figure 7C). Although the three cases show different temporal dynamics, they all approach to an identical one-crypt steady state. In particular, the case *C*_0_ = 1 shows formation of a single crypt without initial TD cells, which is consistent with the experimental observation [34].

**Figure 8:**
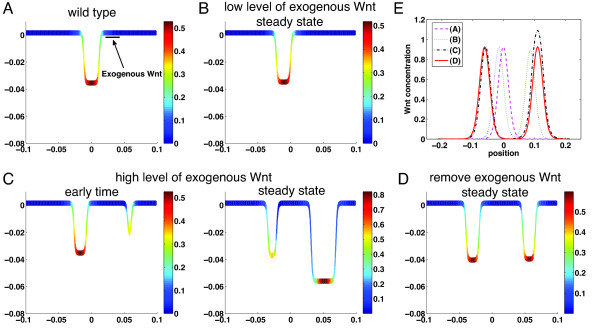
**Exogenous Wnt added at a localized spatial region. (A)** The exogenous Wnt is added at a fixed spatial region of a stable single crypt along the crypt direction $\left[p-\frac{\omega }{2},p+\frac{\omega }{2}\right]$ with a constant rate *θ*. The parameters used for the wild-type single crypt are listed in Additional file 1: Table S2. **(B)** A new stable pattern of a single crypt at a low level of exogenous Wnt: *θ* = 4 × 10^−4^*s*^−1^*μM*. **(C)** A new stable pattern of two-crypt pattern at a high level of exogenous Wnt: *θ* = 4 × 10^−3^*s*^−1^*μM*. **(D)** Removing the exogenous Wnt (i.e. *θ* = 0*s*^−1^*μM*) in steady state of **(C)** leads to formation of two new stable crypts. (E) Wnt patterns at the steady states for **(A)**, **(B)**, **(C)**, and **(D)**. The other parameters are *p* = 0.75*L*_max_, *ω* = 0.05*L*_max_ (*L*_max_ is the crypt length in (A)). The temporal dynamics of the three cases **(B)**, **(C)**, **(D)** are provided in Additional file 1: Figure S6

We next study how local exogenous Wnt may affect the Wnt patterning and formation of crypts. Starting with a stable single crypt, we add exogenous Wnt at a localized region of the crypt (Figure 8A). If the level of exogenous Wnt is low, a similar stable single crypt is formed with a slight shift to the left due to the Wnt (Figure 8B). Because of the periodic boundary conditions used in the model, this suggests a low level of exogenous Wnt may not affect the stable crypt pattern. If the level of exogenous Wnt is high, a second stable crypt, which is larger than the first one, is developed due to the exogenous Wnt (Figure 8C). When the exogenous Wnt is removed from the steady two-crypt system, the newly formed crypt shrinks and two nearly identical crypts are formed at the steady state (Figure 8D). Because progenitor cells can produce Wnt that has its own positive auto-regulation, exogenous Wnt that was initially added and then removed can still induce and sustain growth (Figure 8E). Of course, when too much exogenous Wnt is added, the second crypt is observed to show an uncontrollable growth while the original smaller crypt remains almost unchanged. The overall simulations are consistent with the culture experiment that has shown the exogenous Wnt could substitute the Paneth cells to markedly improve crypt organoid growth [34], suggesting an important role of Wnt and the reaction–diffusion mechanism during cell lineage progression in intestinal crypts.

## Conclusions

An important aspect of colon crypt development and formation is its spatial patterning of cells at different lineage stages within intestinal tissues. Morphogens, signals induced by secreted molecules released from cells, their regulations on cell proliferation and differentiation, and the intimate coupling among signals and cells are generally believed to be the main factors governing growth, dynamics, and maintenance of spatial heterogeneity in crypts.

Here, we focus on spontaneous formation and stability of multiple crypts driven by Wnt and BMP signals that are regulated by cells in the intestinal tissue. Through a model based on a continuum description of two different types of cells in cell lineage with Wnt and BMP molecules produced at different rates by different types of cells, we have demonstrated that Wnt patterning driven by a reaction–diffusion mechanism consisting of short-range activation of Wnt and long-range inhibition by the Wnt inhibitor with additional modulation from BMP could spontaneously result in formation of multiple crypts that are observed experimentally. Unlike typical Turing patterning mechanisms, Wnt signaling in this case is intimately coupled with cell proliferation and expressed in only a portion of cell lineage within the growing tissue.

Our model can recapitulate some distinctive and important experimental facts. First, progenitor cells in crypts can be regenerated during an intestinal injury because Wnt signaling tightly mediates the self-renewal property of progenitor cells, leading to repopulate the original intestinal crypt; in particular, the observation in which new crypts first arise from the existing crypts is captured in the model through a suitable energy functional that mimics the overall mechanistic effects from the surrounding tissues on the crypts. Second, loss of BMP signal leads to crypt multiplication in a form of development of more stable crypts; however, with an additional increase in Wnt signaling during crypt multiplication, uncontrollable growth of crypts is then found in the model simulation – a signature of cancer initiation. The simulations have also shown that crypts exhibit fingering dynamics during crypt multiplications, an interesting pattern consistent with experiments. Model exploration has suggested that Wnt signaling pattern dictates crypt patterning while BMP is mostly responsible for crypt stability. The model presented herein has been developed mainly for studying the role of Wnt and BMP in growth of multiple crypts. In the current model, Wnt positively regulates the progenitor cell replication probability that is also being negatively regulated by BMP. As more molecular details are revealed on specific functions of Wnt and BMP in cell differentiation and proliferation, the model can be extended and refined to incorporate those details.

Because the diffusive molecules, Wnt, Wnt inhibitor, and BMP, may move into the lamina propria, further modulating spatial and temporal patterning of Wnt signaling, hence, affecting crypt organization [35], one may need to add other spatial dimensions beyond the growth direction along the single layer of cells in the model. For the two- or three-dimensional models, the energy function like Eq. (4) needs to be refined to include transport aspects of cells and Wnt and BMP molecules in other spatial directions or different equations for growth of crypts are required based on other mechanistic or phenomenological descriptions.

On the other hand, more intermediate states and cellular types in one or multiple branching cell lineages, which is the case for human colonic crypts, can be added in the current model in a straightforward fashion. With inclusion of more cell types, it would be interesting to investigate effects of symmetric division versus asymmetric division and their interactions with Wnt and BMP signals on crypt patterning. Of course, downstream and feedback regulatory networks of Wnt may be added in the model as well to study functions of target genes of Wnt and BMP (e.g. c-Myc) and their role on cell proliferation or differentiation during growth of colonic crypts, as improperly regulated Wnt signaling results in constitutive renewal and limitless expansion of stem cells or confer stem cell behavior on the progenitor cells, leading to formation of cancerous tissues [5].

Besides Wnt and BMP signaling, the model can be naturally extended to include other important signaling pathways, such as Notch signaling. Notch signaling is expressed in intestinal crypts [36]. Wnt and Notch are found to jointly maintain stem cells with a fact that either Wnt or Notch signaling is insufficient to keep all progenitor cells in a proliferating state [7]. Another avenue of research is why Wnt pathway mutations frequently give rise to intestinal cancers, whereas Notch pathway mutations have not been found to do so. This observation suggests that the stem cells are likely respond to Wnt and Notch signaling differently, for example, with a different regulation in the replication probability or different interactions with BMP or Wnt inhibitors in the model presented in this paper. Another possibility, which can be tested by the model, is that active Wnt signaling may switch on Notch activity, but not vice versa [7].

The cell lineage framework in which cell population is described continuously has its limitation when single cell and small number of cells with strong cell migration and heterogeneity dictate dynamics of the crypt system, which requires the development of a discrete cell lineage model where one needs to trace single stem cell. A recent study by Murray *et al.*[27] has shown that discrete models can be coarsed-grained in a continuum limit to generate a continuum model for more generalized description of the intestinal crypt. In such models, the incompressibility assumption on tissue growth is not required and the tissue compression may be accounted for as well.

In this paper, we have used a phenomenological approach with a simple energy functional describing the morphological change of crypts based on the experimental observation of the shape of crypts. Although the energy functional may be convenient in including certain simple mechanistic effects, incorporating both biochemical and physical mechanisms leading to the complex shape of the crypts requires further development of the continuum models presented in this paper. This more challenging problem is beyond the scope of our current paper and it will be pursued in our future study.

A recent modeling study suggests that the periodic pattern of crypt could be formed by the buckling instability caused by the negative tension produced by the dividing cells [37]. In this model, the epithelia monolayer of cells is assumed to be lying on top of an elastic stroma, and the different patterns of villi and crypts are affected by a coupling of cell division and local curvature. It would be interesting to compare this mechanical mechanism with the chemical mechanism studied in this paper. Because the Wnt patterning is closely related to cell replication probability for cell division, these two mechanisms may be similar in certain aspects in driving the crypt pattern. One possible experimental approach in studying these two mechanisms is to utilize different culture conditions to examine morphological differences of intestinal crypt–villus units built from single stem cell [33]. A mechanical modification of the laminin-rich Matrigel that supports intestinal epithelial growth [33] would lead to pattern changes if the mechanics is the dominant mechanism for crypt patterning. On the other hand, a pulse of exogenous Wnt occurring at different spatially locations [34] would affect Wnt patterns, resulting in different crypt patterning if the reaction–diffusion mechanism presented in this paper is dominant.

## Methods

To solve the system of Eqs. (14), we apply Fourier spectral method to the spatial discretization, and a semi-implicit temporal scheme to the temporal discretization [38]. A periodic boundary condition is chosen for the spatial direction. A typical number of spatial grid points used in the simulations is 256 with a time-step size 10^−4^. Numerical tests have been conducted to ensure sufficient spatial and temporal resolutions for convergence of the numerical solution (see more details in Additional file 1).

## Competing interests

The authors declare that they have no competing interests.

## Authors’ contributions

LZ carried out model development, simulations, analysis, and wrote the manuscript. AL, and QN helped with model development, data analysis, and manuscript. All authors read and approved the final manuscript.

## Supplementary Material

Additional file 1 **A Reaction–diffusion Mechanism Influences Cell Lineage Progression as a Basis for Formation, Regeneration, and Stability of Intestinal Crypts.** Equations for models, numerical methods, stability analysis, tables of parameters, and supplementary figures. Click here for file
