# Supplementary material for: A reaction–diffusion mechanism influences cell lineage progression as a basis for formation, regeneration, and stability of intestinal crypts
Source: BMC Syst Biol. 2012 Jul 31;6:93. doi: 10.1186/1752-0509-6-93 (PMC3434027; doi:10.1186/1752-0509-6-93)
Supplement: Additional file 1 — A Reaction–diffusion Mechanism Influences Cell Lineage Progression as a Basis for Formation, Regeneration, and Stability of Intestinal Crypts. Equations for models, numerical methods, stability analysis, tables of parameters, and supplementary figures. [file 1752-0509-6-93-S1.doc]

**Supporting Material**

**A Reaction-Diffusion Mechanism Influences Cell Lineage Progression as a Basis for Formation, Regeneration, and Stability of Intestinal Crypts**

**Lei Zhang^1,3,4^, Arthur D. Lander^2,3,4^, Qing Nie^1,3,4§^**

^1^Department of Mathematics, University of California, Irvine, CA 92697, USA

^2^Department of Development and Cell Biology, University of California, Irvine, CA 92697, USA

^3^Center for Complex Biological Systems, University of California, Irvine, CA 92697, USA

^4^Center for Mathematical and Computational Biology, University of California, Irvine, CA 92697, USA

^§^Corresponding author

Email addresses:

Lei Zhang: [zhangl4@uci.edu](mailto:zhangl4@uci.edu)

Arthur D. Lander: [adlander@uci.edu](mailto:adlander@uci.edu)

Qing Nie: [qnie@math.uci.edu](mailto:qnie@math.uci.edu)

**Table of Contents**

**I. Cell Lineage Model and Numerical Methods**

**II. Linear Stability Analysis**

**III. Tables**

**IV. Figures**

**V. References**

**I. Cell Lineage Model and Numerical Methods**

*Cell and morphogen equations*

In the cell equation (1), we assume that the volume of substance between cells is negligible and the overall cell population maintains a uniform density, i.e. up to normalization, in both space and time. Then the velocity equation in (1) can be obtained by taking a sum of two equations in (1). Since the TD cells may have higher death rate on the luminal surface, the degradation rate for TD cell is modeled as a function of :

[S1.1]

Here, is the maximal degradation rate of TD cell. is the transition width of decay and is the TD cell concentration at the boundary of luminal surface. In the simulations, we take

In Eq. (3), the convection timescale is due to the tissue growth is and the diffusion timescale is , where L represents the length scale, *V* the average velocity, and *D* is the diffusion rate. The ratio between these two time scales is . The steady state crypt length is , and the stem cell cycle length is between 12 and 32*h* with an average of 24*h* [1]. As a result, the ratio is usually large (e.g., 10.8 in this case) and we can neglect the convection terms in the equations (3). Moreover, because the typical timescales of cell cycle lengths and tissue growth are days, whereas the timescale for molecule interactions is typically hours, the morphogen system quickly reaches steady state with a time scale of cell cycle lengths [2], therefore, we use the quasi-steady-state approximation:

[S1.2]

*Crypt Dynamics*

We use a smooth curve to represent the shape of crypts and spatial dynamics of crypts through gradient of the energy functional [3]. The energy depends on progenitor cells based on the following assumptions:

1. Population of progenitor cells is mostly located at the bottom of crypts, which represents a minimum energy of the crypts.
2. The depth and width of crypts depend on the maximum density and total amount of progenitor cells, respectively.

By using these basic and simple assumptions, we choose two strategies to employ the crypt mechanics:

*Strategy I:* We fix two end points of the crypt and choose the initial crypt (luminal surface) as a flat curve, then one simple form of the energy is

[S1.3]

where is the diameter of crypt which is chosen as a scale of integration of the progenitor cells along a crypt. The parameter is the location of the luminal surface and represents the sharpness of the crypt shape. Two end points are and where .

This strategy is used for simulations in Fig. 2-5, 6A-C, 7, and 8.

*Strategy II:* We can apply the polar coordinate to the energy functional shown in Strategy I:

[S1.4]

We take and  with ( is the ratio of the major axis and minor axis in the ellipse). We use this strategy for crypt multiplication shown in Fig. 6D by choosing .

*Crypt model in growing domain*

The dynamics of the crypt length is governed by the crypt dynamics

,

where is the parametrization of the crypt curve , and are two end points of the curve.

To solve the system of cells and morphogens in growing domain, we first transform Eqs. (S1.1-S1.3) by scaling *s* with such that the new spatial variable is in a fixed domain [-1, 1] and the dynamics of is embedded in the coefficients of the transformed PDEs.

We make the change of variables:

then the transformed cell and morphogen equations (1) and (3) become

[S1.5]

and

[S1.6]

The computational domain is chosen as [,] with for the fixed domain. A periodic boundary condition is chosen in order to account for spatial distribution of cells in periodic multiple crypts. To solve the systems in both fixed domain and growing domain, we apply Fourier spectral method for the spatial discretization, and a semi-implicit scheme is carried out for the temporal discretization [4].

A typical number of spatial grid points used in the simulations are 256 with a time-step size . Numerical tests have been conducted to ensure sufficient spatial and temporal resolutions for convergence of the numerical solution. The Turing pattern of crypt is very robust to the noise in the progenitor cells and the initial Wnt distribution if the removal rate of Wnt is low (Fig. S7). For high removal rate of Wnt, the number of crypts at steady state may be varied for different initial distribution of Wnt (Fig. 3).

In our one-dimensional models, we focused on cell movement and dynamics of Wnt and BMP along the crypt direction without incorporating details of the geometry and growth of the crypt. To incorporate dynamic growth and geometry of crypt, one needs to modify Eqs (1-4) by replacing the independent variable by the dynamic arc-length variable where is the independent parametrization variable in a fixed domain (e.g. [0,1]). Now the derivatives with respect to in Eqs (1-4) become

with being updated in time through Eq. (4). The new overall system for one-dimensional growth model then can be solved by using the similar computational approach in this paper.

**II. Linear Stability Analysis**

To investigate the conditions of Turing instability for molecules Wnt and Wnt inhibitor, we apply the method in [5] to study the first two equations in [S1.4]:

[S2.1]

[S2.2]

Assuming ,,, which greatly simplifies the calculation, the system is reduced to

[S2.3]

where .

In order to simplify the equation further, we take , which are the numbers used for all simulations and figures, and we normalize the system with a new scale:

Then Eq. [S2.3] in the new variables becomes

[S2.4]

where

First, the homogeneous steady-state solution is

The perturbation of this solution takes the form:

with

Following the method in [5], for the general system

the conditions for the generation of spatial Turing patterns at the steady state are

The derivatives and must be of opposite sign. Thus, we have

Since and must have opposite signs, it requires . With these expressions, the conditions of instability require

[S2.5]

Notice that , we have

[S2.6]

By setting , the phase diagram of Turing instability region (Fig. S1) shows that, in addition to the requirement that Wnt inhibitor diffuses faster than Wnt, Wnt inhibitor has to adapt rapidly to any change of the Wnt, which corresponds to the case that the ratio between removal rate and satisfies a constraint, i.e., . In addition, the ratio of the two diffusion coefficients between Wnt inhibitor and Wnt determines the ratio of the corresponding removal rates for Turing instability that is necessary for formation of heterogeneous pattern.

**III. Tables**

Table S1: Parameters used for Fig. 2-8 and Fig. S1-S6 unless otherwise specified ‘--’ means “not applicable”.

| Parameters | Value | Units |
| --- | --- | --- |
| ,, | ,, |  |
| , | , |  |
|  | 1 | -- |
| , | 2, 2, 2, 1, 2 | -- |
| , | 4, 10 |  |
|  | 0.25 | Per cell cycle |
|  | 0.05 | Per cell cycle |
|  | 120, 0.1 |  |
|  | 0.2, , 0.1 |  |
|  | 0.6 |  |
|  | 0.002 |  |
|  | 0.1 |  |

**Table S2: Parameters used in Fig. 2-8 and Fig. S1-S6 ‘--’ means “not applicable”.**

| Parameters | () | () | () |
| --- | --- | --- | --- |
| Fig. 2 |  |  |  |
| Fig. 3 | -- | -- |  |
| Fig. 4A |  |  | -- |
| Fig. 4B-C |  |  |  |
| Fig. 5 |  |  |  |
| Fig. 6A-D |  |  |  |
| Fig. 6E |  |  |  |
| Fig. 7, 8 |  |  |  |
| Fig. S2 |  |  |  |
| Fig. S3 |  |  |  |
| Fig. S4 |  |  |  |
| Fig. S5 |  |  |  |
| Fig. S6 |  |  |  |

**IV. Figures**


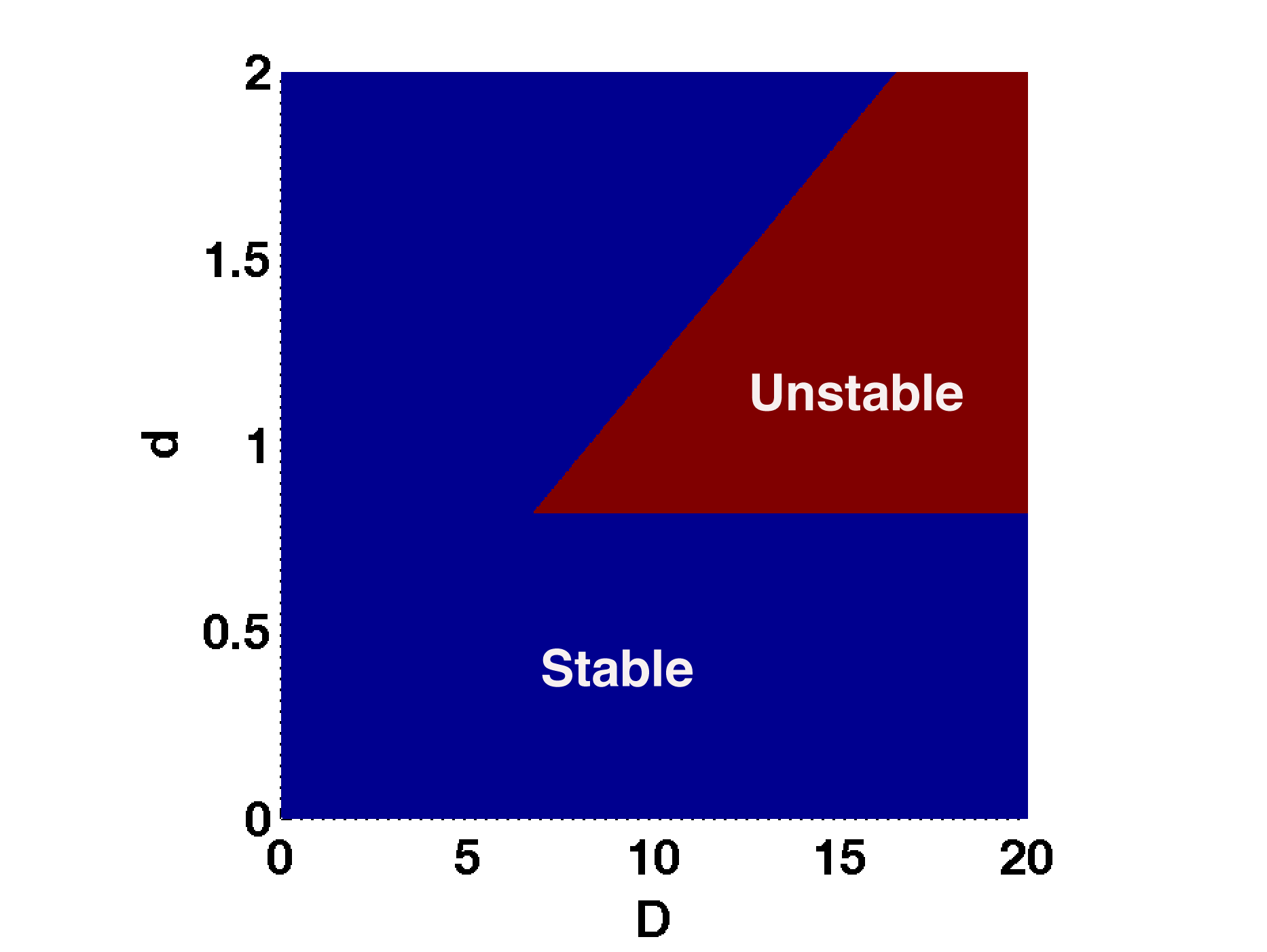


**Figure S1. Phase diagram of Turing instability.** For generating Turing instability, the ratio of the two diffusion coefficients between Wnt inhibitor and Wnt determines the ratio of the corresponding removal rates. “Unstable” means the region satisfying the conditions of Turing instability; “Stable” represents the region not satisfying the conditions of Turing instability. x-axis: , y-axis: .


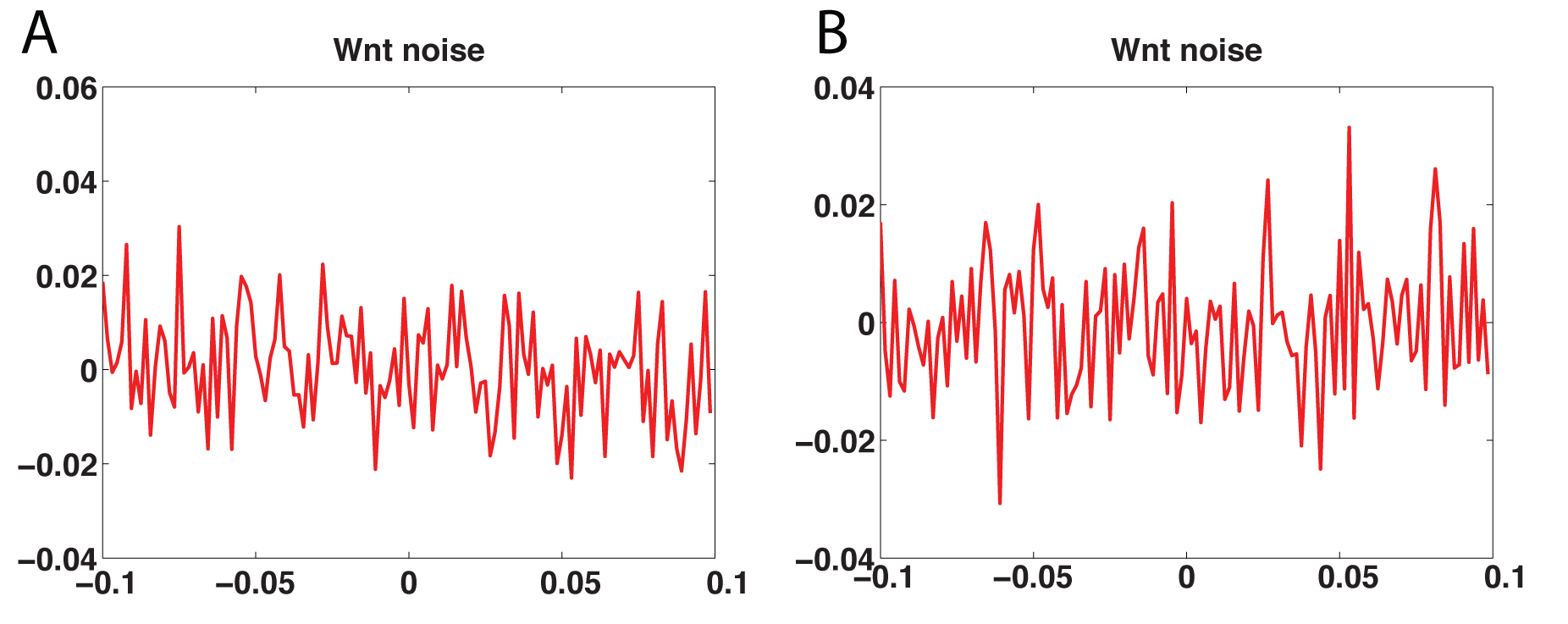


**Figure S2. Initial conditions of Wnt at the removal rate of Wnt** **for Figure 3 in main text.**

(A) Initial condition for five crypts at the steady state;

(B) Initial condition for six crypts at the steady state.


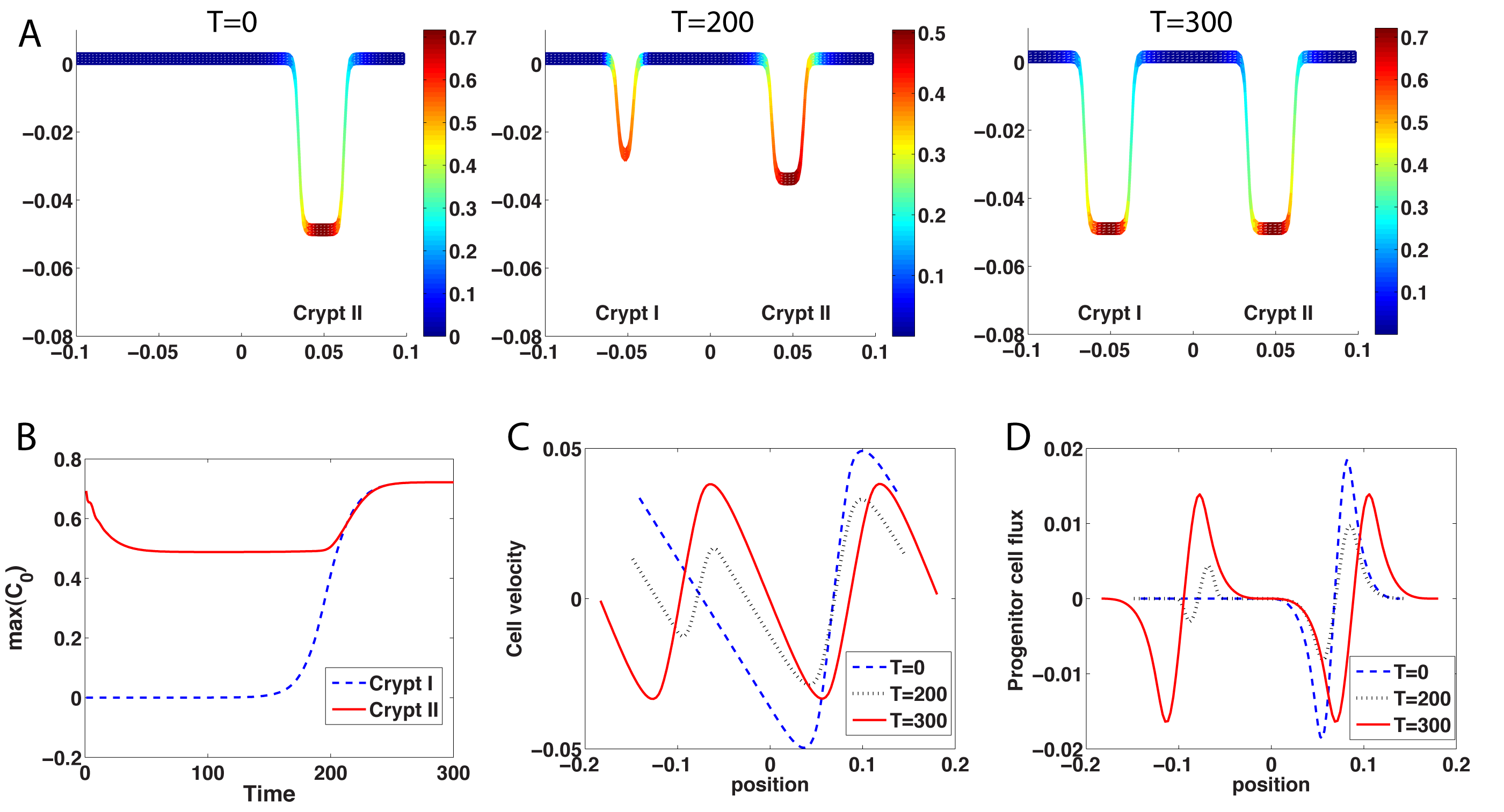


**Figure S3: Dynamics of crypt regeneration when Crypt I was removed.**

(A) At , all progenitor cells in Crypt I are removed from the wild-type steady crypt ( in Fig. 2A). Progenitor cells traveling from Crypt II start to accumulate and self-renew, leading to formation of new Crypt I (). All progenitor cells in both crypts are ultimately regenerated (). (B) The maximal density of progenitor cells in Crypt I (blue dash line) and Crypt II (red solid line) as functions of time. Cell velocity, , (C) and progenitor cell flux, , (D) are plotted at the time *T*=0 (blue dash curve), *T*=200 (black dot curve), and *T*=300 (red solid curve).

**
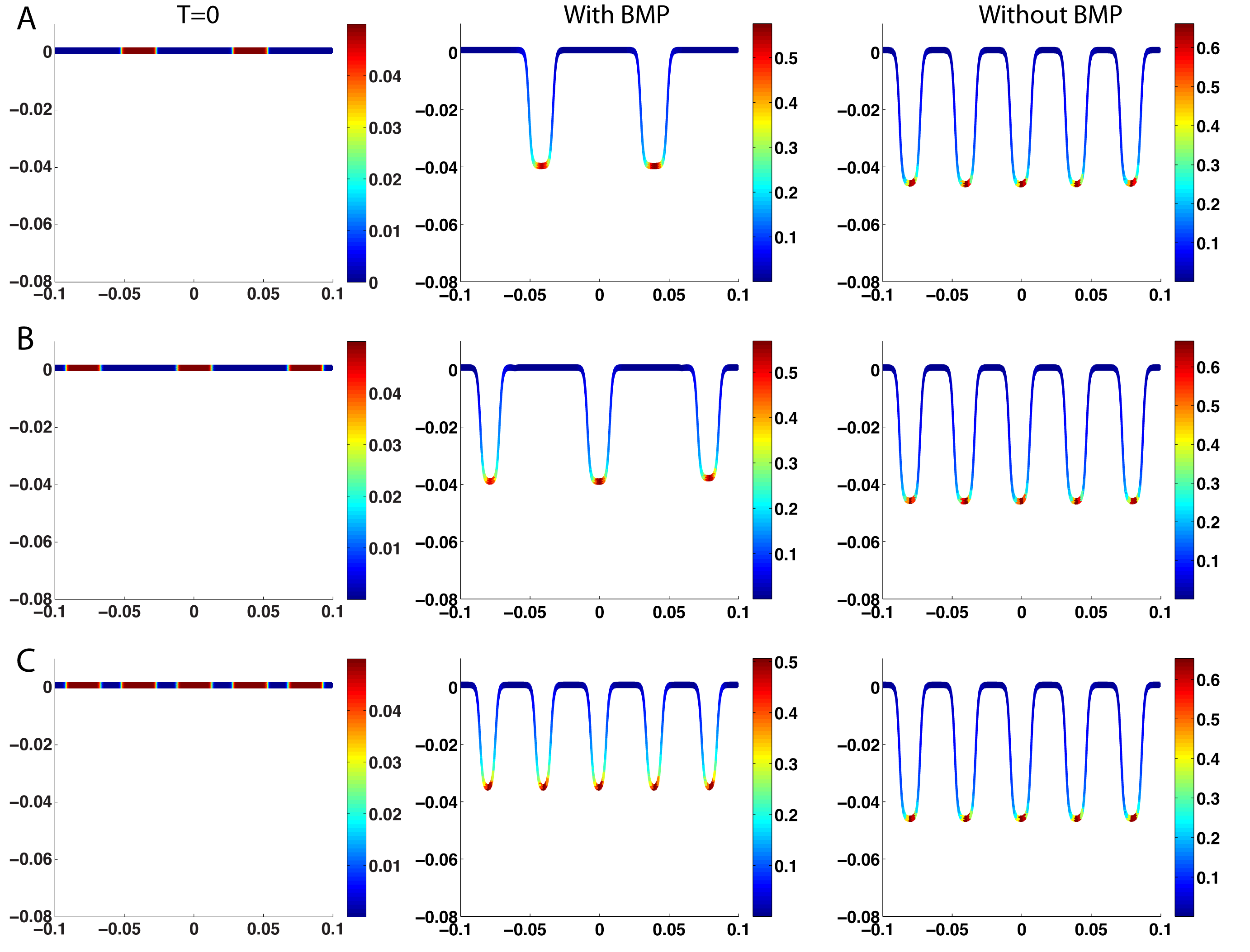
**

**Figure S4. For a system with BMP, different initial distributions of progenitor cells result in different multiple steady state crypts. However, for the system without BMP, the same five-crypt steady state is always generated at the steady state for all the three initial conditions.**

(A) Two localized spots of the progenitor cells at , leading to two steady crypts with BMP and five steady crypts without BMP.

(B) Three localized spots of the progenitor cells at , leading to three steady crypts with BMP and five steady crypts without BMP.

(C) Five localized spots of the progenitor cells at , leading to five steady crypts with BMP and five steady crypts without BMP.


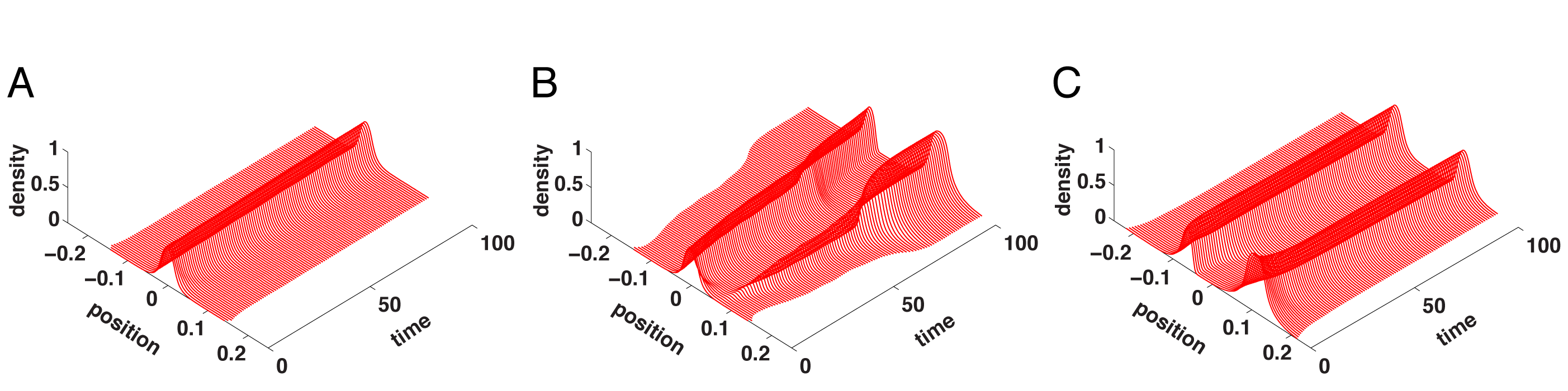


**Figure S5: Temporal dynamics of the progenitor cells by adding exogenous Wnt.**

(A) Temporal dynamics of Fig. 10 (B) at ,

(B) Temporal dynamics of Fig. 10 (D) at ,

(C) Temporal dynamics of Fig. 10 (E) at .


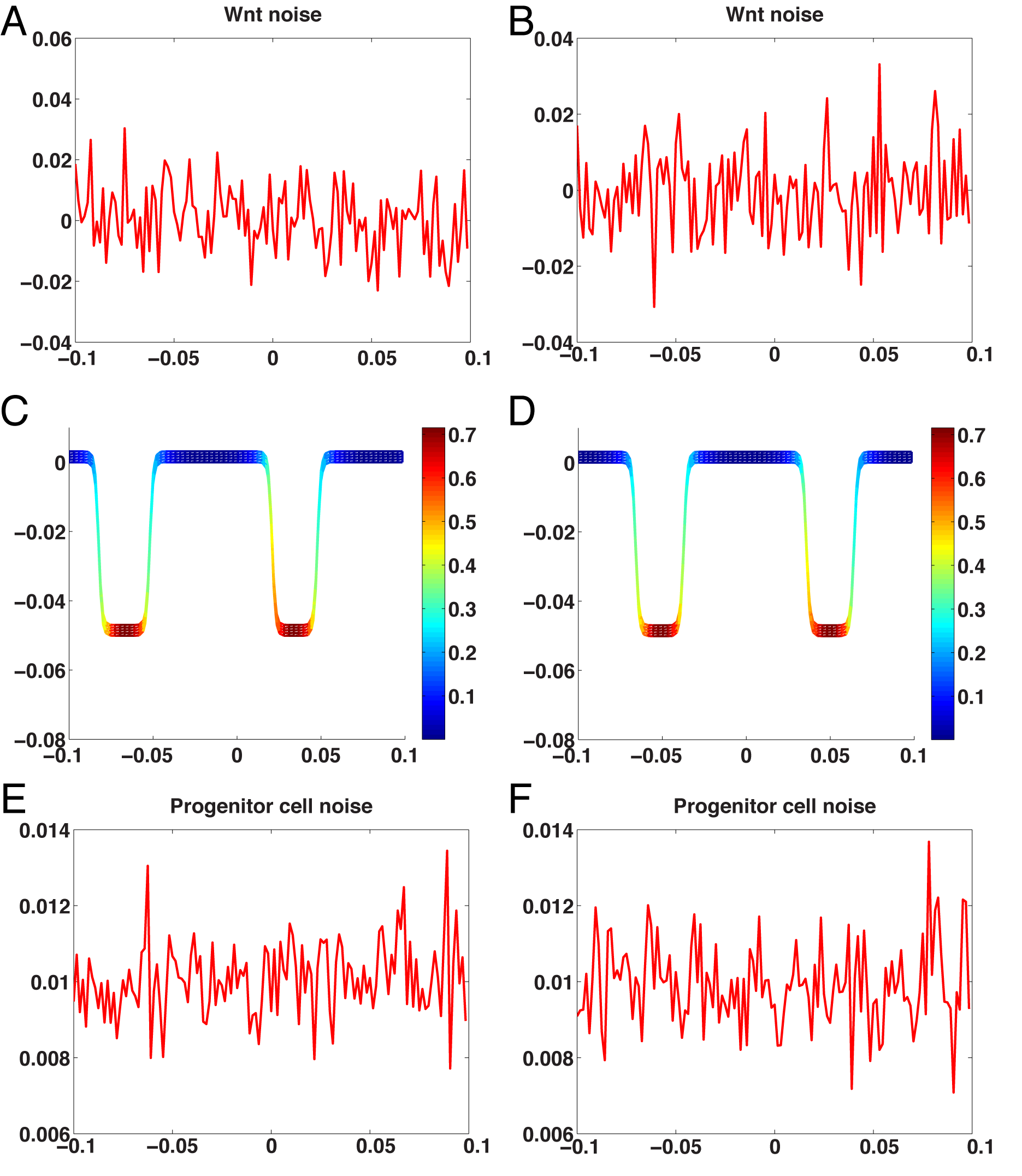


**Figure S6: Noise effect on the formation of crypt pattern.** Taking different noises in the initial Wnt at (A) and (B), it results in the same periodic two-crypt pattern (C) and (D), respectively. The crypt formation is also very robust to the noise in the progenitor cells. Two samples of progenitor cells noise are plotted in (E) and (F).


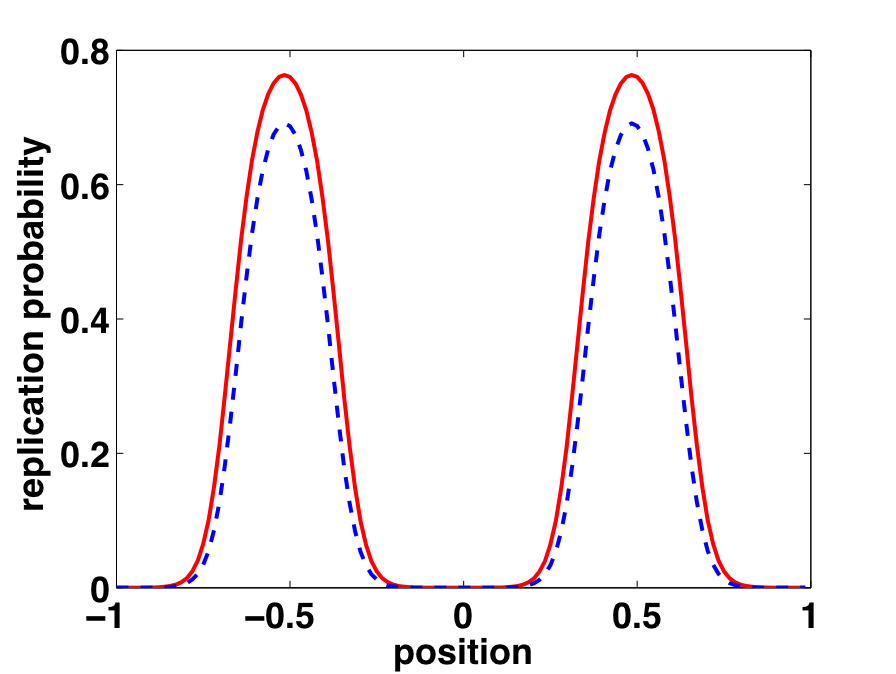


**Figure S7:** Replication probabilities of progenitor cells with the maximal death rate of TD cell (red solid curve) and (blue dash curve).

V. References

1. Johnston M, Edwards C, Bodmer W, Maini P, Chapman S: Mathematical modeling of cell population dynamics in the colonic crypt and in colorectal cancer. *Proceedings of the National Academy of Sciences* 2007, 104:4008.

2. Chou CS, Lo WC, Gokoffski KK, Zhang YT, Wan FY, Lander AD, Calof AL, Nie Q: Spatial dynamics of multistage cell lineages in tissue stratification. *Biophys J* 2010, 99:3145-3154.

3. Evans LC: *Partial Differential Equations.* American Mathematical Society; 1998.

4. Chen L-Q, Shen J: Applications of semi-implicit Fourier-spectral method to phase field equations. *Computer Physics Communications* 1998, 108:147-158.

5. Murray JD: *Mathematical Biology.* Springer-Verlag, Berlin; 1993.
